# Genome Wide Association Analysis of a Founder Population Identified *TAF3* as a Gene for MCHC in Humans

**DOI:** 10.1371/journal.pone.0069206

**Published:** 2013-07-31

**Authors:** Giorgio Pistis, Shawntel U. Okonkwo, Michela Traglia, Cinzia Sala, So-Youn Shin, Corrado Masciullo, Iwan Buetti, Roberto Massacane, Massimo Mangino, Swee-Lay Thein, Timothy D. Spector, Santhi Ganesh, Nicola Pirastu, Paolo Gasparini, Nicole Soranzo, Clara Camaschella, Daniel Hart, Michael R. Green, Daniela Toniolo

**Affiliations:** 1 Division of Genetics and Cell Biology, San Raffaele Research Institute and Vita Salute University, Milano, Italy; 2 Cardiovascular Research Institute, University of California San Francisco, San Francisco, California, United States of America; 3 Wellcome Trust Sanger Institute, Hinxton, Cambridge, United Kingdom; 4 Novi Ligure Hospital, Novi Ligure (Al), Italy; 5 Department of Twin Research & Genetic Epidemiology, King's College London, London, United Kingdom; 6 Department of Molecular Hematology, King’s College London, London, United Kingdom; 7 Division of Cardiovascular Medicine, University of Michigan Health System, Ann Arbor, Michigan, United States of America; 8 Medical Genetics, Department of Reproductive Sciences and Development, University of Trieste, Trieste, Italy; 9 Medical Genetics, Department of Laboratory Medicine, Institute for Maternal and Child Health IRCCS-Burlo Garofolo, Trieste, Italy; 10 Howard Hughes Medical Institute, Program in Gene Function and Expression and Molecular Medicine, University of Massachusetts Medical School, Worcester, Massachusetts, United States of America; 11 Institute of Molecular Genetics-CNR, Pavia, Italy; University of Turin, Italy

## Abstract

The red blood cell related traits are highly heritable but their genetics are poorly defined. Only 5–10% of the total observed variance is explained by the genetic loci found to date, suggesting that additional loci should be searched using approaches alternative to large meta analysis. GWAS (Genome Wide Association Study) for red blood cell traits in a founder population cohort from Northern Italy identified a new locus for mean corpuscular hemoglobin concentration (MCHC) in the *TAF3* gene. The association was replicated in two cohorts (rs1887582, *P* = 4.25E–09). *TAF3* encodes a transcription cofactor that participates in core promoter recognition complex, and is involved in zebrafish and mouse erythropoiesis. We show here that TAF3 is required for transcription of the *SPTA1* gene, encoding alpha spectrin, one of the proteins that link the plasma membrane to the actin cytoskeleton. Mutations in S*PTA1* are responsible for hereditary spherocytosis, a monogenic disorder of MCHC, as well as for the normal MCHC level. Based on our results, we propose that *TAF3* is required for normal erythropoiesis in human and that it might have a role in controlling the ratio between hemoglobin (Hb) and cell volume and in the dynamics of RBC maturation in healthy individuals. Finally, *TAF3* represents a potential candidate or a modifier gene for disorders of red cell membrane.

## Introduction

Erythrocytes comprise 40–50% of blood volume and are key components for the transport of oxygen and carbon dioxide for cellular respiration. In healthy individuals the red blood cell-related traits (hemoglobin (Hb), hematocrit (Hct), red cell count (RBC), mean corpuscular volume (MCV), mean corpuscular hemoglobin (MCH) and mean corpuscular hemoglobin concentration (MCHC)) are variable and are determined by both inherited and environmental factors. Besides age and sex, acquired factors such as diet, smoking, body weight, hypoxia, blood loss, infections and inflammation are well known to influence erythropoiesis. Heritability of red blood cell traits is high (h2 = 04 to 06) [1] but the genetic determinants remain largely unknown. A better knowledge of the inherited factors determining variation of erythrocyte traits will be relevant to understand the physiology of red blood cell production and the relationship between red cell size and Hb content. This might have implication also for the so-called anemia of the elderly [2], [3], [4] contributing to cognitive impairment and impaired physical capacity in older people.

Recently, 75 independent genetic loci associated with red blood cell phenotypes have been reported from a meta analysis including >135.000 individuals of European or South Asian ancestry. Together, they explain 4–9% of the phenotypic variance per trait [5]. Among the different approaches considered, the use of selected populations, such as genetically isolated populations, was suggested to account for the remaining variability [6] and help in better defining the genetic component of complex traits. Because of their recent origin from a limited number of founders and of subsequent isolation, such populations have been predicted to facilitate the identification of rare variants in the general population that may be enriched in a founder population or could be more easily detected in association with common variants thanks to increased linkage disequilibrium (LD). The relatively homogeneous genetic background and environmental exposure are well-established characteristics of genetic isolates [7], [8], [9], [10]. Moreover, comparison with environmental effects and collection of longitudinal data over several decades may facilitate investigation of relationships between genetic variation and studied traits, early life events and progression of disease risks.

We have collected health and family data of an Italian isolated population living in a valley of North West Italy in the Apennine Mountains, the Val Borbera (INGI-VB) inhabited by about 3000 descendants from the original population [1], [11]. GWAS of the available red blood cell traits identified a novel locus on chromosome 10, associated with MCHC. The locus encompassed the *TAF3* (TATA box binding protein (TBP)-associated factor 3) gene, previously shown to be involved in erythropoiesis in zebrafish and mouse [12]. We present here evidence for a role of TAF3 in expression of cytoskeletal proteins relevant for RBC membrane size and for MCHC in humans.

## Materials and Methods

### Study subjects

The INGI-VB population [1] is described in Materials S1. Only individuals aged 18 years or older were eligible to participate in the study.

The cohorts used in the study (one group of genetically isolated populations from North East Alps in Italy, the Friuli Venezia Giulia (INGI-FVG) region, and outbred population-based cohorts of European ancestry, TwinsUK, AGES, RS, FHS and InChianti) are described in Materials S1.

### Blood tests

Fasting blood samples were obtained in the early morning. Complete blood cell (CBC) count was performed the same day by the diagnostic laboratory of the Novi Ligure (AL, Italy) Hospital, by standard methods [1]. MCHC (mean corpuscular hemoglobin concentration) is expressed as grams of hemoglobin per 100 ml of packed cells and is calculated as the ratio between hemoglobin concentration and hematocrit.

### Genotyping and association analysis

One thousand six hundred and sixty-four DNAs from the INGI-VB population were genotyped using the Illumina 370 Quad-CNV array, v3. 343,867 SNPs passed quality control checks (SNP call rate >90%, MAF ≥1%, HWE P-value ≥10–4) and were used in subsequent studies. We also imputed genotypes from the 2.5 millions polymorphic HapMap data set, using MACH (http://www.sph.umich.edu/csg/abecasis/mach/). We chose an estimated r^2^ >0.3 as a threshold to flag and discard low-quality imputed SNPs.

GWAS of the red blood cell traits was done using the GenABEL and ProbABEL packages [13] that take into account the relatedness among the INGI-VB cohort, using genomic kinship. In order to focus on determinants of normal variation of hematological traits in the general populations we restricted the analysis to those individuals within three standard deviations of the population mean. To normalize the distributions, natural log transformation was applied to MCH, MCHC and MCV and square root transformation was applied to RBC, prior to analysis. We used an additive model including sex and age as covariates for Hb, Hct, MCH, MCV, RBC and sex and age^2^ for MCHC. Quantile-Quantile (Q-Q) plots of all the GWAS are shown in Fig. S1.

For meta-analysis we used a fixed-effects inverse variance method as implemented in METAL (http://www.sph.umich.edu/csg/abecasis/Metal/index.html).

### Cell culture and transfections

Mouse erythroleukemia (MEL) cells [14] were a kind gift from Merav Socolovsky {University of Massachusetts Medical School}. K562 cells were obtained from the American Type Culture Collection [ATCC® CCL-243]. MEL cells were maintained in Dulbecco’s Modified Eagle’s medium (DMEM) supplemented with 10% fetal bovine serum (FBS). Erythroid differentiation was induced by the addition of dimethyl sulfoxide (DMSO) to 2% in culture medium. Human K562 cells were maintained in RPMI supplemented with 10% FBS.

### ShRNA- induced knockdowns

Two anti human TAF3 shRNA constructs, TRCN0000016610, and TRCN0000016612, were obtained from The RNAi Consortium (Broad Institute). Two anti-mouse TAF3 shRNA constructs, SM2441e7, and SM2496h12 and the control scrambled shRNA were obtained from Open Biosystems (Huntsville, AL, USA). These constructs were electroporated into cells using the Amaxa Nucleofector AAD-1001 electroporator (Lonza, Walkersville, MD, USA). Cell line Nucleofector Kit V (Cat. No. VCA-1003) was used according to manufacturer’s instructions (Lonza, Walkersville, MD, USA).

### Chromatin immunoprecipitation (ChIP)

ChIP was performed using standard protocols, with the following modifications: sequential cross-linking was performed using ethylene glycol bis[succinimidyl succinate] (EGS) and formaldehyde as follows. Cells were crosslinked in 1.5 mM EGS in PBS for 20 minutes at room temperature, followed by the addition of formaldehyde to 1% final concentration for 10 minutes. Affinity purified rabbit anti-TAF3 was obtained from Bethyl Laboratories (Montgomery, TX, USA), catalog No. A302–360A. Isolated chromatin was sheared using a Diagenode Bioruptor (Liege, Belgium). Fold enrichment was calculated as normalized fold differences of cycle thresholds [2^–(−ΔΔCt)]^ from specific IP over non specific IgG after quantitative PCR.

### Quantitative RT-PCR

Total RNA was isolated and reverse transcription performed, followed by quantitative real-time PCR using SYBR Green on an 7500 Fast Real Time PCR System (Applied Biosystems-Life Technologies Corporation, Carlsbad, CA, USA). For all reactions, the Ct values of samples were analyzed after subtracting the signal obtained with the non-silencing shRNA (for RNAi) controls. Fold knockdown or gene expression level was calculated by normalizing the expression of the target gene following knockdown to that obtained using a non-silencing shRNA. TAF3 recruitment levels were determined by normalizing levels to those obtained with no antibody controls.

The primers used are listed in Tables S1 and S2.

### Ethics Statements

INGI-VB. The study was approved by San Raffaele Hospital and Regione Piemonte ethical committees. Written informed consent was obtained from every participant to the study as indicated by the San Raffaele Hospital and Regione Piemonte ethical committees.

INGI-FVG. Ethics approval was obtained from the Ethics Committee of the Burlo Garofolo Children Hospital in Trieste. Written informed consent was obtained from every participant to the study as indicated by the Burlo Garofolo Children Hospital Ethics Committee.

TWINSUK. Twins largely volunteered unaware of any their phenotypic status in relationship to SAA or any other phenotypic trait of interest to the authors and they gave written fully informed consent under a protocol reviewed and approved by the St Thomas’ Hospital Local Research Ethics Committee.

AGES. The AGES-Reykjavik Study GWAS was approved by the National Bioethics Committee (VSN: 00–063) and the Data Protection Authority. Written informed consent was obtained from every participant to the study as indicated by the National Bioethics Committee

FHS. The research protocols of the Framingham Heart Study are reviewed and approved annually by the Institutional Review Board of the Boston University Medical Center and by the Observational Studies Monitoring Board of the National Heart, Lung and Blood Institute. Since 1971, written consent has been obtained from participants before each examination.

InCHIANTI. The ethical committee of the INRCA “I Fraticini,” Florence, Italy, approved the InCHIANTI study protocol. All participants signed an informed participation consent.

## Results

### Identification of a new locus for MCHC in the INGI-VB population

GWAS of haemoglobin concentration and related parameters was performed in 1664 genotyped individual from INGI-VB and many of the SNPs previously identified [15], [16], [17] as associated to RBC related traits were replicated. Some of the SNPs represented nominally significant replicas, others had a highly significant p-value (Table S3). In this analysis we also identified two novel GW significant loci, both associated with MCHC (Table 1). One, rs1155865 on chromosome 4 (p-value  = 3.05E–08), was in a region devoid of any known gene A group of genes could be found only more than 1 Mb down stream. This association could not be replicated. The second SNP, rs1887582, on chromosome 10 was borderline GW significant (p-value  = 3.75E–07). The presence of the variant significantly decreased MCHC from an average value of 33.13 g/dl in AA homozygotes to 32.65 g/dl in GG homozygotes. The association of the SNP rs1887582 could be replicated in two additional cohorts (INGI-FVG and Twins UK) (Table 2). The combined p-value was highly significant (beta  = −0.0059, SE  = 0.0010, p-value  = 4.25E–09, HET p-value  = 0.591) (Fig. S2). No other red blood cell trait was significantly associated with rs1887582.

**Table 1:** SNPs associated with MCHC in the INGI-VB cohort.

Trait	SNP	Chr	Position (Build 36)	P-value	Other allele	Effect allele	Effect allele freq	N	Effect	SE	% VAR
MCHC	rs1155865	4	67416452	3.05E–08	A	G	0.15	1653	−0.0089	0.0016	1.90
MCHC	rs1887582	10	8043339	3.75E–07	A	G	0.22	1655	−0.0069	0.0014	1.56

**Table 2:** rs1887582 replica.

Cohort	P-value	Other allele	Effect allele	Effect allele freq	N	Effect	SE
INGI-VB	3.75E–07	A	G	0.22	1655	−0.0069	0.0014
TWINSUK	9.63E–03	A	G	0.17	3396	−0.0050	0.0020
INGI-FVG*	2.51E–02	A	G	0.16	619	−0.0047	0.0021

* Illegio-Sauris-Resia.

We could study four additional cohorts (RS, FHS, AGES and InChianti) : rs1887582 variant was significantly associated in AGES (p-value  = 8.70E–04). The other populations did not show association: in all three, the variant had an opposite sign effect (Table 3).

**Table 3:** rs1887582 association in general population cohorts.

Cohort	P-value	Other allele	Effect allele	Effect allele freq	N	Effect	SE
AGES	8.70E–04	A	G	0.17	3184	−0.0021	0.0006
RS	1.69E–02	A	G	0.18	5381	0.0020	0.0009
FHS	1.26E–01	A	G	0.18	3166	0.0012	0.0008
inChianti	8.26E–01	A	G	0.17	1018	0.0003	0.0021

### The new MCHC locus encompasses the *TAF3* gene

**Figure 1:**
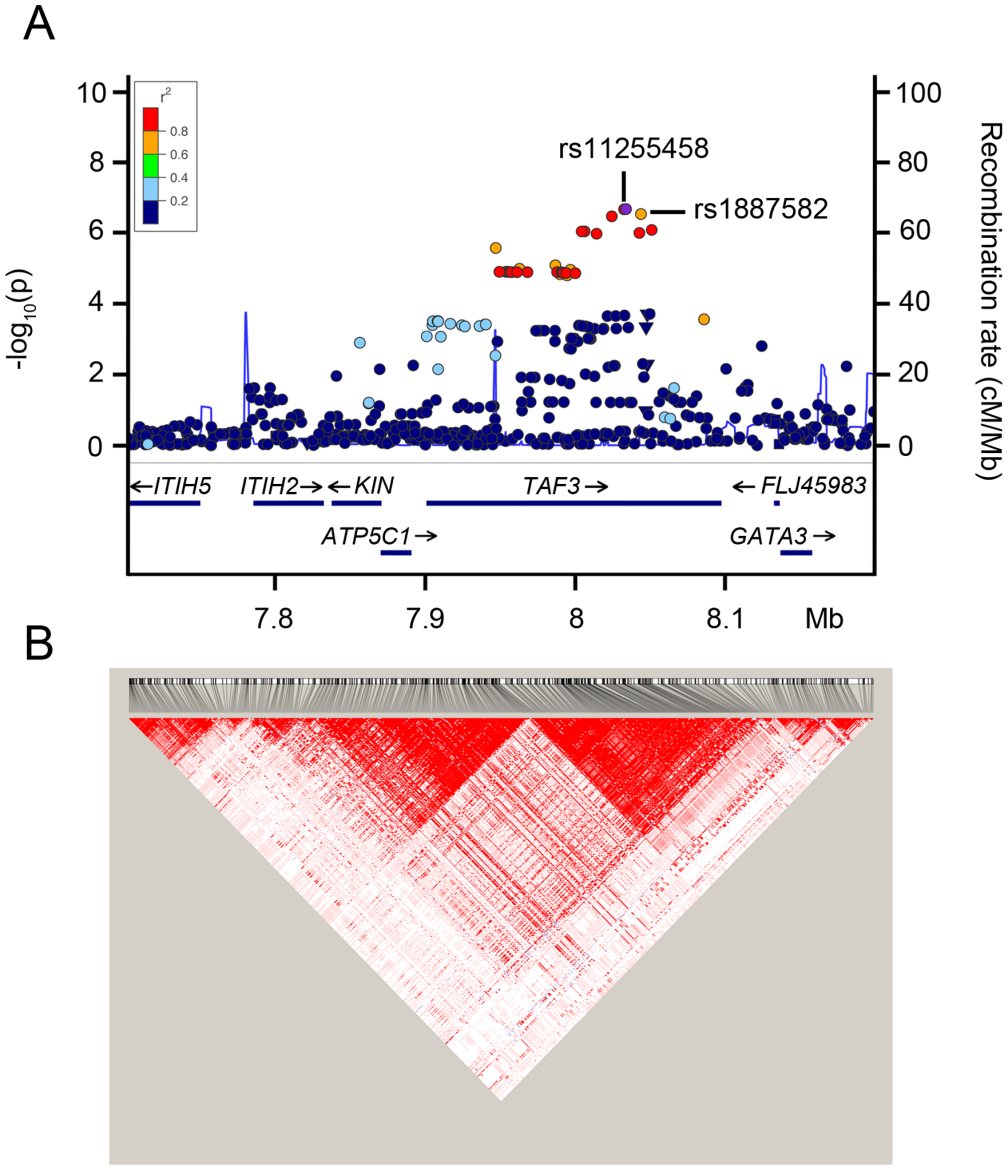
Regional association plot and linkage disequilibrium pattern at the TAF3 locus. (A) Association of genotyped and imputed SNPs. The top imputed SNP (rs11255458) is highlighted in violet, the other SNPs are colored according to their degree of linkage disequilibrium (r^2^) with rs11255458. The chromosomal positions (NCBI human genome Build 36) of the SNPs are plotted against genomic control-adjusted -log10 p-value. The estimated recombination rates (cM/Mb) from HapMap CEU release 22 are shown as gray lines. (B) The D'-based LD map was build using genotyped and imputed data of the INGI-VB population.

The SNP rs1887582 mapped to the *TAF3* gene (Fig 1). A number of additional SNPs, in LD with rs1887582 appeared associated with MCHC when the imputed SNP dataset was analyzed. The imputed SNP rs11255458 presented the lowest p-value (beta  = −0.0073, SE  = 0.0014, p-value  = 2.28E–07) and had an r^2^  = 0.88 with rs1887582. The peak of association mapped within the TAF3 gene. The recombination and LD map of the region are shown in Fig. 1A (CEU cohort from HapMap) and 1B (INGI-VB cohort). One recombination hot spot, separating two LD blocks and the variant from the promoter of the gene, was localized in the middle of the gene, proximal to association peak. Association analysis of MCHC conditioned on rs11255458 did not identify independent signals and confirmed that the association is due to LD to the SNP rs11255458.

Interestingly, in K562 erythroid cells the locus is enriched for the histone modification H3K4me1, a histone mark associated with enhancer regions (Fig. S3). The rs11255458 SNP introduces a C/T change in the binding site for a repressor transcription factor, YY1, as determined by TESS analysis of the sequence [CACACAC/TTT) in both C and T forms ( http://www.cbil.upenn.edu/cgi-bin/tess/tess ). YY1 may direct histone deacetylases and histone acetyltransferases to promoter /enhancer regions. A C/T mutation in YY1 binding site may thus disrupt or enhance the erythrocyte-specific transcription of *TAF3*.

### TAF3 interacts with the *SPTA1* promoter and is required for its regulated expression

**Figure 2:**
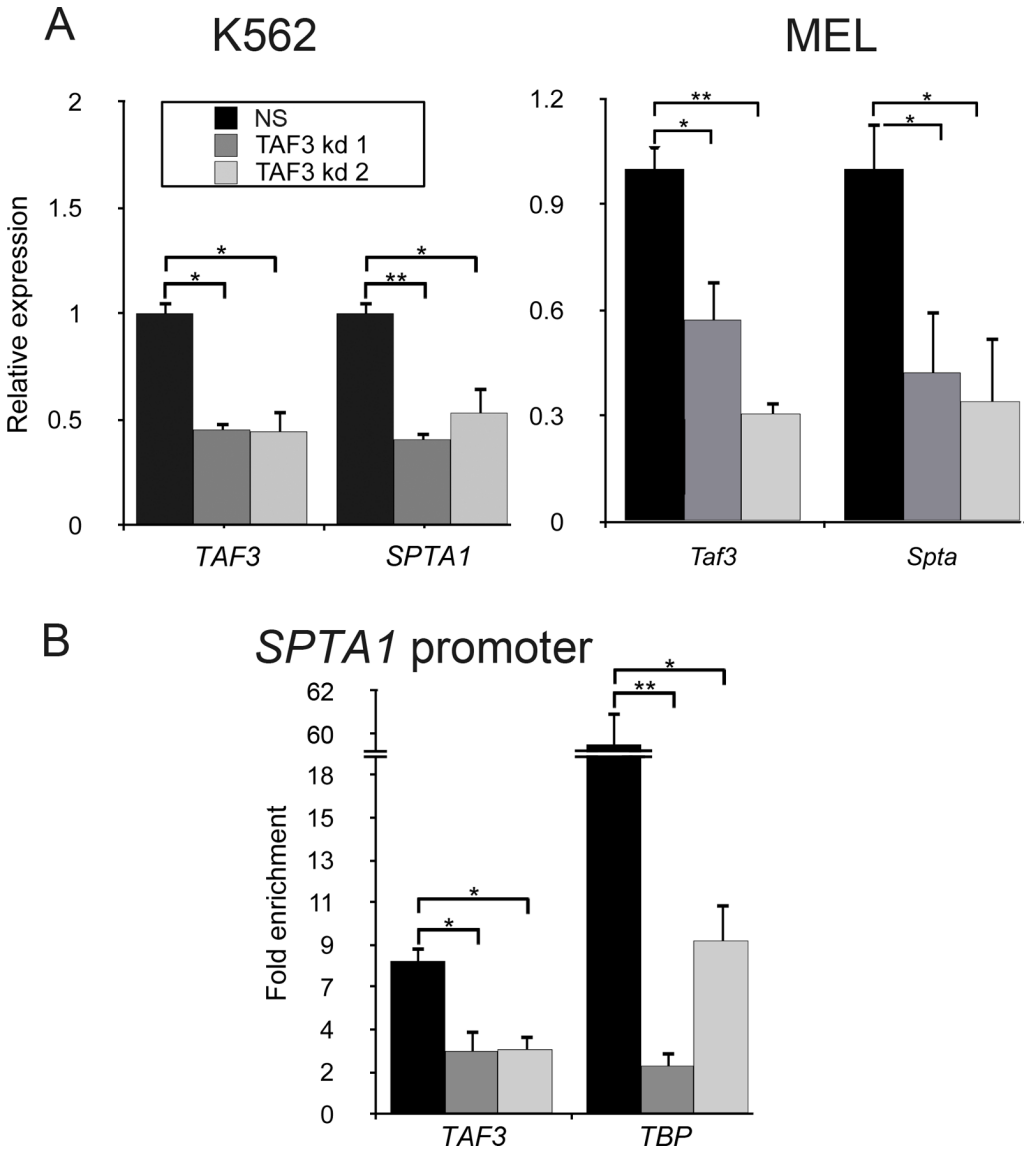
The *SPTA1* gene is transcriptionally regulated by *TAF3*. (A) Knockdown of *TAF3* by shRNA and its effect on *SPTA1* expression in human K562 cells, and in differentiating mouse MEL cells. 4 days after induction. Expression was monitored by qRT-PCR. NS: control scrambled shRNA; *TAF3*kd1 and *TAF3* kd2 and anti *Taf3* shRNAs described in Methods. (B) Chromatin immunoprecipitation analysis of the recruitment of *TAF3* and *TBP* to the indicated promoters in K562 cells shows that *TAF3* is associated with *SPTA1*. *TAF3*: ChIP with anti Taf3 Ab; IgG: control chromatin immunoprecipitation with IgG addition. Asterisks indicate a statistical significant difference (*  = *P*<0.05; **  = *P*<0.01) between control and TAF3 knockdown data for MEL and K562 cells, and between specific immunoprecipitations and IgG control immunoprecipitations.

MCHC, the ratio between Hb and Hct, may be altered by changes in membrane structure that modify the surface/volume ratio of the erythrocyte, as observed in hereditary spherocytosis. *TAF3* encodes a member of the large family of cofactors that specifically interact with TRF3 (TBP-related factor 3), one of the TATA binding proteins (TBP). We tested whether *TAF3* could influence MCHC through transcriptional regulation of *SPTA*1, a gene encoding alpha spectrin, a red cell protein that links the plasma membrane to the actin cytoskeleton and is one of the genetic determinant of MCHC in disease [18] and in the normal range[15] (see also Table S3). We analyzed the expression of the gene in human K562 cells expressing shRNAs designed to target the *TAF3* transcript. We observed a 50% reduction in the level of *SPTA1* expression, by qRT-PCR analysis, in cell treated with both shRNAs compared with control scrambled shRNA(NS-shRNA) (Fig. 2A). We also transiently introduced constructs bearing control or *Taf3* shRNAs into mouse erythroleukemia MEL cells. Transfected MEL cells were induced to erythroid differentiation for 4 days, and then monitored for the expression of *SPTA1* by qRT-PCR analysis. As shown in Fig. 2 A, knockdown of *TAF3* strongly reduced the expression of the mouse gene as well.

To determine whether TAF3 directly regulates transcription of *SPTA1*, we performed chromatin immunoprecipitation assays in K562 cells and examined TAF3 and TBP recruitment to the *SPTA1* promoter. We observed significant recruitment of TAF3 and TBP in NS shRNA K562 cells. The observed reduction in TBP recruitment to the SPTA1 promoter following TAF3 knockdown [Fig 2b] is consistent with the known stable association of TAF3 and TBP as components of the TFIID complex [19], [20]. These data confirm a role for TAF3 in regulating the transcription of *SPTA1*.

## Discussion

We report here a new locus, associated with MCHC levels identified by GWAS of red blood cell traits in the INGI-VB founder population. The association to decreased MCHC level of the minor allele could be replicated in other populations, where it did not reach GW significance, confirming that isolated populations may be relevant tools for the identification of loci that escape large meta-analysis. Variants, rare in the general population, may have drifted to higher frequency in the genetic isolate, while the recent origin from a limited number of founders could have produced increased LD and homogenous LD maps. The SNP rs1887582 was indeed found at a 30% higher frequency of 0.22 in INGI-VB compared to 0.17 of most other cohorts. The presence of a recombination hot spot in the middle of the genetic association peak could have produced several different haplotypes in outbred populations, and increased the genetic heterogeneity. A unique haplotype in the isolated INGI-VB population, resulting from the less heterogeneous genetic background, could account for the differences in the p-values of the association in the different cohorts. Finally, the observation that the rs11255458 SNP introduces a C/T change in the binding site for a repressor transcription factor, YY1 in a putative enhancer region suggests that it could be the genetic background of the isolate that allows the identification of the variant as causative of an increase in MCHC.

The novel locus highlighted a transcription factor, *TAF3*, essential in hematopoiesis in zebrafish and in the mouse [12]. *TAF3* encodes a member of the large family of cofactors that specifically interact with TRF3, one of the TATA binding proteins (TBP) and participates in recruitment of the multi-subunit core promoter recognition complex [12], [21]. We present here evidence for a role of this gene in human, in expression of proteins relevant for RBC membrane organization. The red cell has to maintain its structural integrity, membrane stability and deformability that allow erythrocyte survival in the circulation, especially in the spleen, in order to assure oxygen delivery to the tissues. Membrane deformability is mainly achieved by maintenance of vertical links between cytoskeleton components and the red cell membrane lipid bilayer. Weakening these links leads to decreased membrane surface, increased cell volume and increased MCHC. The role of cytoskeletal proteins in determining normal MCHC variation was confirmed by the finding of DNA variation(s) in *SPTA1* associated with MCHC in the normal population [15] and in our sample (table S3). Mutations in *SPTA1* were found in the monogenic disorder of MCHC, hereditary spherocytosis (HS, MIM ID #182900). Our results in K562 and MEL cells demonstrate a direct role for *TAF3* in the transcriptional control of SPTA1, and bring further insights into the molecular pathways controlling red blood cell cytoskeleton.

*TAF3* variation does not affect other RBC traits. We propose that it might be specifically involved in control of RBC volume and ultimately in the regulation of the ratio between Hb and cell volume and in the dynamics of RBC maturation in normal individuals. The mechanisms that regulate number, size and Hb concentration in normal erythropoiesis are poorly understood. Our results shed some light in a process continuously taking place in normal individuals, as ∼2.5E–11 new RBC are released every day from the bone marrow in the peripheral circulation to replace those that are continuously cleared and this number can increase up to 20 fold in cases of severe anemia [22], [23].

Finally, as mutations in various membrane and cytoskeleton components have been described in disorder affecting RBC geometry and particularly cell surface to volume ratio, TAF3 may represent a candidate gene for rare cases of HS or other inherited red cell membrane defects, non linked to known genes or a modifier gene for rare cytoskeleton disorders affecting the severity of disease phenotype.

## Supporting Information

Figure S1 **Quantile-quantile plots for the traits indicated.** The x axis shows -log10 transformed expected P values, while the y axis indicates -log10 transformed observed P values. The corresponding genomic inflation factor (λ) is also shown for each trait.
(DOC)

Figure S2 **Forest plots of effect size and direction for rs1887582.** The contributing effect from each study is shown by a black square, with confidence intervals indicated by horizontal lines. The contributing weight of each study to the meta-analysis is indicated by the size of the square. The combined meta-analysis estimate is shown at the bottom of each graph.
(DOC)

Figure S3 **Graphical representation of the TAF3 gene intron where the rs112355458 SNP maps.** The rs112355458 SNP is indicted by the red arrow. The locus is enriched for the histone modification H3K4me1, a histone mark associated with enhancer regions. The rs11255458 SNP may disrupt the erythrocyte-specific transcription of *TAF3* by generating a binding site for a repressor complex.
(DOC)

Materials S1 (DOC)

Table S1 **Primers for gene expression analysis in MEL cells.**
(DOC)

Table S2 **Primers for ChIP analysis in K562 cells.**
(DOC)

Table S3 **Comparison of the GWAS results of the present study with previous studies.**
(DOC)
