# Supplementary figures and images for: Genome Wide Association Analysis of a Founder Population Identified TAF3 as a Gene for MCHC in Humans
Source: PLoS One. 2013 Jul 31;8(7):e69206. doi: 10.1371/journal.pone.0069206 (PMC3729833; doi:10.1371/journal.pone.0069206)

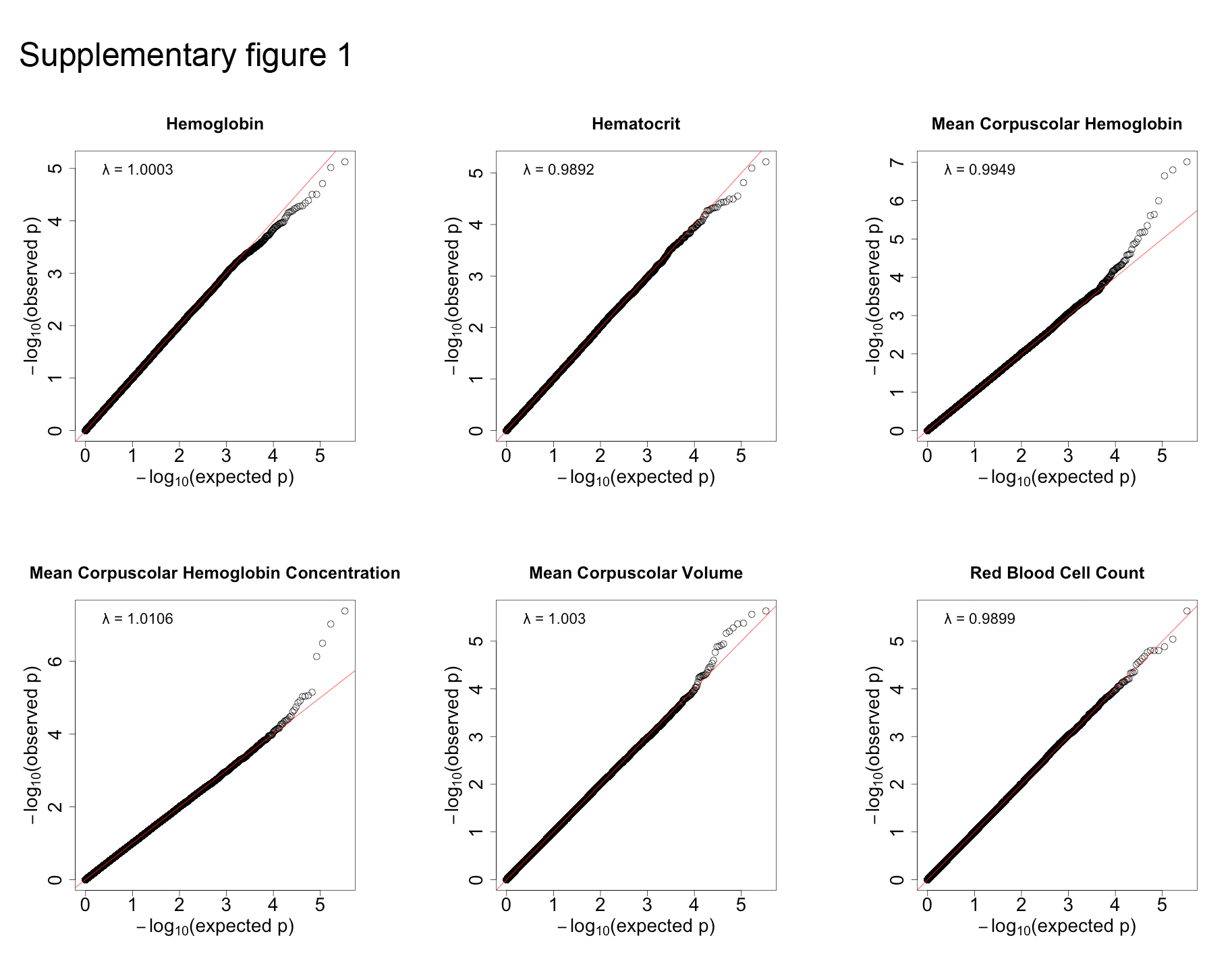


Figure S1

Supplement: Figure S1 — Quantile-quantile plots for the traits indicated. The x axis shows -log10 transformed expected P values, while the y axis indicates -log10 transformed observed P values. The corresponding genomic inflation factor (λ) is also shown for each trait. (DOC) [file pone.0069206.s001.doc]

Figure S2


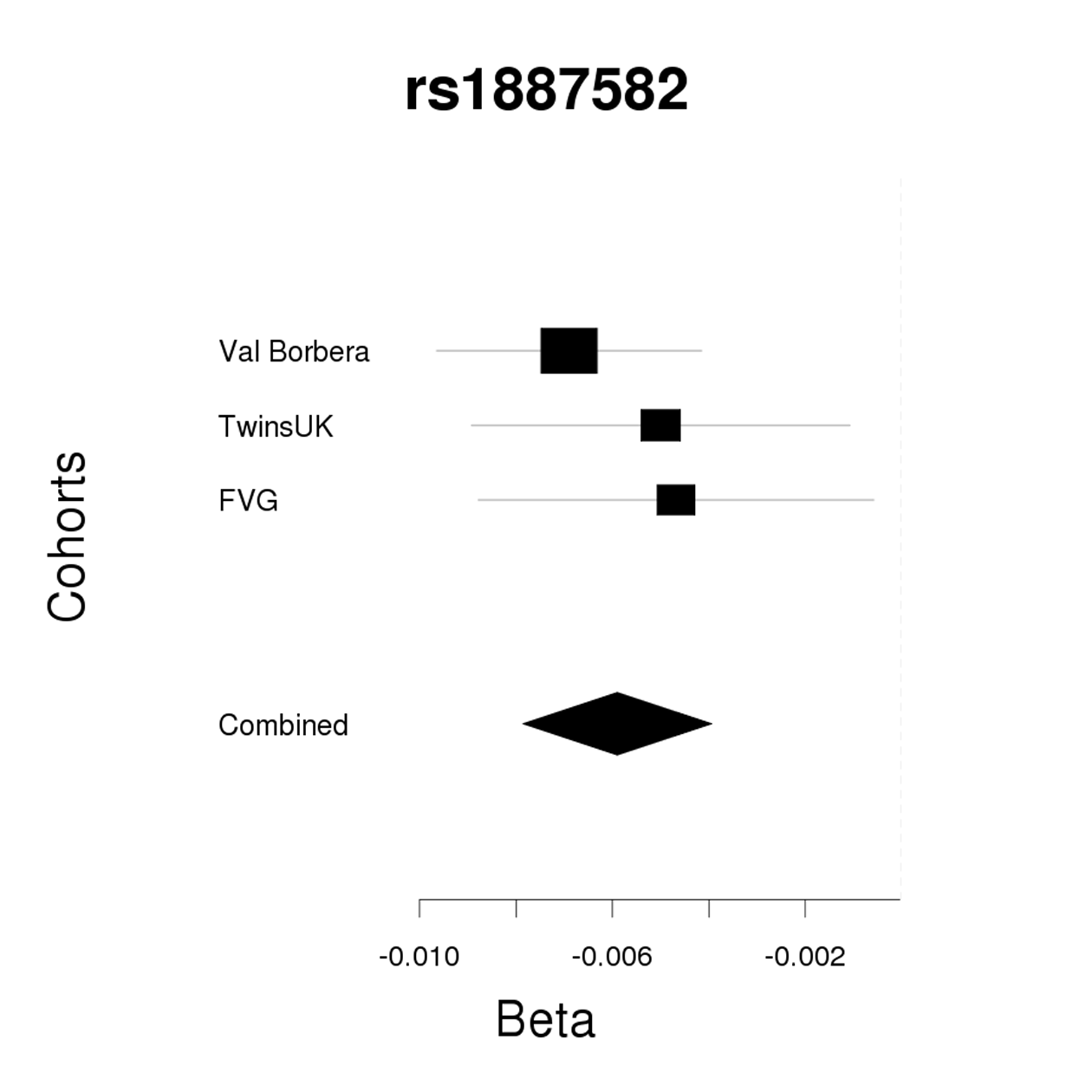

Supplement: Figure S2 — Forest plots of effect size and direction for rs1887582. The contributing effect from each study is shown by a black square, with confidence intervals indicated by horizontal lines. The contributing weight of each study to the meta-analysis is indicated by the size of the square. The combined meta-analysis estimate is shown at the bottom of each graph. (DOC) [file pone.0069206.s002.doc]

Figure S3


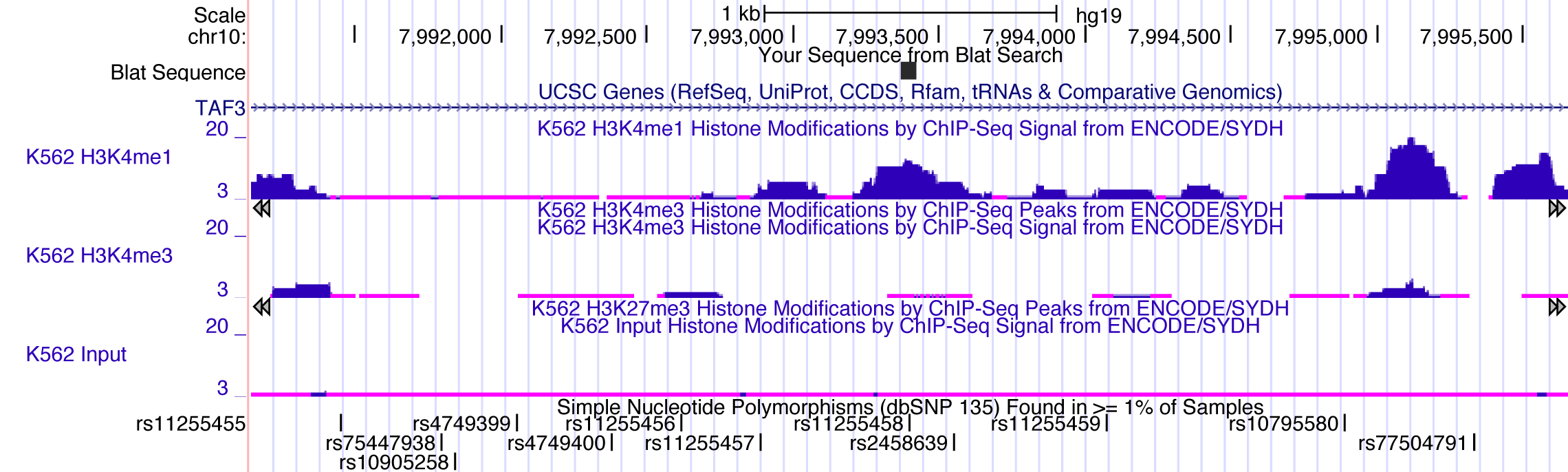

Supplement: Figure S3 — Graphical representation of the TAF3 gene intron where the rs112355458 SNP maps. The rs112355458 SNP is indicted by the red arrow. The locus is enriched for the histone modification H3K4me1, a histone mark associated with enhancer regions. The rs11255458 SNP may disrupt the erythrocyte-specific transcription of TAF3 by generating a binding site for a repressor complex. (DOC) [file pone.0069206.s003.doc]
