# Supplementary material for: Genome Wide Association Analysis of a Founder Population Identified TAF3 as a Gene for MCHC in Humans
Source: PLoS One. 2013 Jul 31;8(7):e69206. doi: 10.1371/journal.pone.0069206 (PMC3729833; doi:10.1371/journal.pone.0069206)
Supplement: Materials S1 — (DOC) [file pone.0069206.s004.doc]

**SUPPLEMENTAL FILES**

**STUDY SAMPLES**

**Val Borbera (INGI-VB)**

*Study subjects.* The INGI-VB population is a collection of 1664 genotyped samples collected in the Val Borbera Valley, a geographically isolated valley located in the Appennine Mountains in NorthWest Italy. The Val Borbera cohort is part of the Italian Genetic Isolates Network (INGI). The valley is inhabited by about 3000 descendants from the original population, living in 7 villages along the valley and in the mountains. The valley was inhabited by about 10,000 people in the 19th century when endogamy was > 80% . Around 1930, the population started to decrease due to emigration to South America. Participants were healthy people between 18 and 102 years of age that had at least one grandfather living in the valley. The study was approved by San Raffaele Hospital and Regione Piemonte ethical committees. The health status of the population was assessed as reported (Traglia et al, 2009).

*Blood measurements.* Fasting blood samples were obtained in the early morning. Blood was tested the same day or aliquoted and stored at -80°C for further analysis. All the tests were done in the laboratory of ASL 22 - Novi Ligure (AL). Blood cell counts and erythrocyte indexes were determined using either an SF3000 haematology analyzer or a XE2100 haematology analyser. The two instruments displayed no significant statistical differences in measurements range. Hb was measured and reported in g/dL, Hct is reported as a percentage, MCH is reported in picograms, MCHC is reported as g/dL, MCV is reported in femtoliters and RBC is reported as millions of cells per cubic millimeter.

**Friuli-Venezia Giulia (INGI-FVG)**

*Sample.* The FVG cohort is comprehends about 1300 samples from an 5 isolated villages of Friuli Venezia Giulia a region of northern Italy. The FVG cohort is part of the Italian Genetic Isolates Network (INGI). The villages include Resia, Illegio and Sauris. Genotyping data for 619 adults is available. Mean age of the partecipants was 51.7 (SD 16.4). Because of the particular structure of this cohort we used both molecular kinship and the village as covariate for the statistical analysis. Ethics approval was obtained from the Ethics Committee of the Burlo Garofolo children hospital in Trieste. Written informed consent was obtained from every participant to the study.

*Genotyping and imputation****.*** All samples were typed with illumina 370k CNV chip (Illumina, San Diego, USA). Imputation of genotypes was carried out using the software MACH. The following QC filters were applied prior to imputation: call rate > 95% , MAF ≥ 1% , HWE P-value ≥ 10^-4^ and per SNP call rate ≥ 90%. 276,271 SNPs where then used as backbone for the imputation. The total number of SNPs used for the analysis was 2,599,375.

**TwinsUK.**

The TwinsUK adult twin registry based at St Thomas’ Hospital in London is a volunteer cohort of over 10,000 twins recruited among the general population. Twins largely volunteered unaware of any their phenotypic status in relationship to SAA or any other phenotypic trait of interest to the authors and they gave fully informed consent under a protocol reviewed by the St Thomas’ Hospital Local Research Ethics Committee. All participants in the TwinsUK study were female and SAA measurements were obtained as described in the main methods section.

*Genotyping* of the TwinsUK dataset was done with a combination of Illumina arrays (HumanHap300^1,2^, HumanHap610Q, 1M-Duo and 1.2MDuo 1M). We pooled the normalised intensity data ^3^ for each of the three arrays separately (with 1M-Duo and 1.2MDuo 1M pooled together). For each dataset we used the Illluminus calling algorithm [4](http://www.plosgenetics.org/article/info%3Adoi%2F10.1371%2Fjournal.pgen.1000445#pgen.1000445-Teo1) to assign genotypes in the pooled data. No calls were assigned if an individual's most likely genotyped was called with less than a posterior probability threshold of 0.95. Validation of pooling was achieved via a visual inspection of 100 random, shared SNPs for overt batch effects. Finally, intensity cluster plots of significant SNPs were visually inspected for over-dispersion biased no calling, and/or erroneous genotype assignment. SNPs exhibiting any of these characteristics were discarded.

*Data QC.* We applied similar exclusion criteria to each of the three dataset separately. *Samples*: Exclusion criteria were: (i) sample call rate <98%, (ii) heterozygosity across all SNPs ≥2 s.d. from the sample mean; (iii) evidence of non-European ancestry as assessed by PCA comparison with HapMap3 populations; (iv) observed pairwise IBD probabilities suggestive of sample identity errors; (v). We corrected misclassified monozygotic and dizygotic twins based on IBD probabilities. *SNPs*. Exclusion criteria were (i) Hardy-Weinberg p-value<10^−6^, assessed in a set of unrelated samples; (ii) MAF<1%, assessed in a set of unrelated samples; (iii) SNP call rate <97% (SNPs with MAF≥5%) or < 99% (for 1% ≤ MAF < 5%). Alleles of all three datasets were aligned to HapMap2 or HapMap3 fwd strand alleles.

*Data merge*. Prior to merging, we performed pairwise comparison among the three datasets and further excluded SNPs and samples to avoid spurious genotyping effects, indentified as follows: (i) concordance at duplicate samples <1%; (ii) concordance at duplicate SNPs <1%; (iii) visual inspection of QQ plots for logistic regression applied to all pairwise dataset comparisons; (iv) Hardy-Weinberg p-value<10^−6^, assessed in a set of unrelated samples; (v) observed pairwise IBD probabilities suggestive of sample identity errors. We then merged the three datasets, keeping individuals typed at the largest number of SNPs when an individual was typed at two different arrays. The merged dataset consists of 5,654 individuals (2,040 from the HumanHap300, 3,461 from the HumanHap610Q and 153 from the HumanHap1M and 1.M arrays) and up to 874,733 SNPs depending on the dataset (HumanHap300: 303,940, HumanHap610Q: 553,487, HumanHap1M and 1.M: 874,733).

*Imputation.* Imputation was performed using the IMPUTE software package (v2) ^5^ using two reference panels, P0 (HapMap2, rel 22, combined CEU+YRI+ASN panels) and P1 (610k+, including the combined HumanHap610k and 1M reduced to 610k SNP content).

***CHARGE Consortium***

The Cohorts for Heart and Aging Research in Genetic Epidemiology (CHARGE) consortium1 includes five cohort studies that have genotyped high density SNP markers and have phenotypic data on erythrocyte traits. We have additionally incorporated data from the InCHIANTI Study for these analyses. Representatives from each constituent cohort formed a hematology working group. The group was responsible for phenotype harmonization, covariate selection, analytic plans for within‐study analyses, meta‐analysis of results and reporting of results. Each participating study was reviewed and approved by the corresponding IRB, and all subjects used for genetic analyses provided specific informed consent for genetic research. All participating studies approved guidelines for this collaboration, including data sharing and data security procedures.

*Age, Gene/Environment Susceptibility‐Reykjavik Study (AGES).* The AGES‐Reykjavik Study cohort originally comprised a random sample of 30,795 men andwomen born in 1907‐1935 and living in Reykjavik in 1967. A total of 19,381 people attended, resulting in 71% recruitment rate. The study sample was divided into six groups by birth year and birth date within month. One group was designated for longitudinal follow up and was examined in all stages. One group was designated a control group and was not included in examinations until 1991. Other groups were invited to participate in specific stages of the study. Between 2002 and 2006, the AGES‐Reykjavik study re‐examined 5764 survivors of the original cohort who had participated before in the Reykjavik Study. The AGES‐Reykjavik Study GWAS was approved by the National Bioethics Committee (VSN: 00‐063) and the Data Protection Authority. Hb, Hct, MCH, MCHC, MCV and RBC were measured in all participants of the AGESReykjavik Study. The parameters were measured in fasting whole blood on a Beckman Coulter (HMX). Between assay quality control procedures were used and the coefficient of variation of the method for each component was 1.10%, 0.84%, 1.42% for RBC, Hb and Hct, respectively. For the erythrocyte indices the coefficient of variation of the method was 0.72%, 1.08% and 1.32% for MCV, MCH and MCHC respectively. Before removal of individuals outside of the +/‐ 3 SD range, there were 3218 subjects, and exclusion on this basis led to the removal of 27 subjects for Hb, 16 for Hct, 39 for MCH, 22 for MCHC, 30 for MCV, 13 for RBC. The final sample size is as in (ref red blood cell paper).

For AGES, DNA was genotyped in 3,219 participants who were eligible for this study using the Illumina 370CNV BeadChip array. Samples were excluded from the dataset based on sample failure, genotype mismatch with reference panel, and sex mismatch. Standard protocols for working with Illumina data were followed. Prior to genotype imputation, SNPs were excluded using filters based on call rate (<97%), Hardy‐Weinberg Equilibrium (<1e‐6), mishap (<1e‐9), and mismatched positions between Illumina, dbSNP and/or HapMap resulting in 325,094 SNPs passing all QC (of 353,202 prior to cleaning steps). Imputation was done using MACH against all the HapMap CEPH haplotypes (release 22/NCBI build 36) resulting in 2,533,153 total SNPs for analysis.

*Rotterdam Study (RS)* The RS is a prospective population‐based cohort study that addresses determinants and occurrence of cardiovascular, neurological, ophthalmologic, psychiatric, and locomotor diseases in the elderly8, 15. For the first cohort, all residents of Ommoord, a suburb of Rotterdam, aged 55 years and over were invited to participate. A total of 7,983 men and women entered the study (response rate 78 percent). Baseline data were collected from 1990 until 1993. Trained research assistants interviewed the participants at home. A total of 7,129 participants visited the research centre twice for a physical examination.

Venous blood samples were drawn from non‐fasting participants. Hb, Hct, MCH, MCHC, MCV and RBC were assessed immediately after blood collection with an automated blood cell counter (Coulter Counter T660) using Beckman reagents. After exclusion of participants with values outside the 3 SD range, participants with complete genetic and phenotypic information were 5,475 for Hb, 5,466 for Hct, 5,405 for MCV, 5,378 for MCHC, 5,413 for MCH, and 5,523 for RBC. Plated DNA was available for 6,680 (83.7%) of 7,129 participants who visited the research center. Genotyping was conducted using the Illumina 550K array among self‐reported Caucasian individuals, and succeeded in 6,240 individuals (sample call rate ≥ 97.5%). We excluded subjects for excess autosomal heterozygosity, mismatch between called and phenotypic gender, or being outliers identified by the IBS clustering analysis. The final population for genetic analysis comprised 5,974 subjects. SNPs were excluded for minor allele frequency ≤1%, Hardy‐Weinberg equilibrium *P* value<10‐6, or SNP call rate ≤90% resulting in data on 530683 SNPs.

*Framingham Heart Study (FHS).* The FHS is a prospective cohort study that aims to identify the risk factors for cardiovascular disease. Under the direction of the National Heart, Lung, and Blood Institute (NHLBI), the FHS recruited 5,209 men and women, the Original Cohort from the town of Framingham, Massachusetts in 1948; 5,124 of the second generation of the Original Cohort, the Offspring Cohort in 1971; and 4,095 participants of the Third Generation in 2002. The FHS cohort studied here is the Offspring Cohort, whose first exam was completed in 1975. For FHS, Hb, Hct, MCH, MCHC, MCV and RBC were measured at Exam 1 of the Offspring Cohort taken place between 1971 and 1975, as previously described5, 13, 14. Blood was collected and spun at 5,000 rpm for 20 min in a balanced oxalate tube. Hematocrit was measured by the Wintrobe method. The percent of total blood volume that was due to RBCs was determined visually against a calibrated scale. Before removal of individuals outside of the +/‐ 3SD range, there were 3,200 subjects for all red blood cell indices except 3,381 for Hct and exclusion on this basis led to the removal of 21 subjects for Hb, 22 for Hct, 57 for MCH, 34 for MCHC, 56 for

MCV, 21 for RBC. DNA was genotyped for the FHS participants with the Affymetrix 500K array and an additional gene‐focused 50K array. MACH software was used to impute ~2.5 million SNPs based on the HapMap CEU phased haplotypes (build 22) and the SNPs that met the following criteria: minor allele frequency (MAF) >=0.01, Hardy Weinberg Equilibrium *P* value >10‐6, SNP call rate >= 97%, MISHAP test *P* value >10‐9, Mendelian errors <= 100. Linear mixed effects model was used to account for familial correlation in the FHS GWAS.

*The Invecchiare in Chianti (InCHIANTI).* The InCHIANTI Study is a population‐based epidemiological cohort study in the Chianti region of Tuscany, Italy. The study employs two clinical sites, in the towns of Greve and Bagno a Ripoli (study site was used as an additional covariate in all analyses), with participants recruited from the population registries of these immediate areas. Further details on this cohort has been previously published elsewhere7. Overnight fasted blood samples were used for genomic extraction and hematological analyses at baseline. Hematological assays were carried out using a Coulter LH 750 (Beckman Coulter, Instrumentation Laboratory, Milan, Italy), with fasting blood sample aliquots stored at ‐ 80 Celsius and thawed at time of assay. DNA extracted from InCHIANTI participants was genotyped at the Laboratory of Neurogenetics, National Institute on Aging, using Illumina 550K beadchips. After standard QC measures, genotypes were imputed using MACH. Maximum likelihood genotypes were filtered for quality of imputation prior to analysis.

**Rotterdam Study (RS).** The RS is a prospective population‐based cohort study that addresses determinants and occurrence of cardiovascular, neurological, ophthalmologic, psychiatric, and locomotor diseases in the elderly8, 15. For the first cohort, all residents of Ommoord, a suburb of Rotterdam, aged 55 years and over were invited to participate. A total of 7,983 men and women entered the study (response rate 78 percent). Baseline data were collected from 1990 until 1993. Trained research assistants interviewed the participants at home. A total of 7,129 participants visited the research centre twice for a physical examination.

Venous blood samples were drawn from non‐fasting participants. Hb, Hct, MCH, MCHC,

MCV and RBC were assessed immediately after blood collection with an automated blood cell counter (Coulter Counter T660) using Beckman reagents. After exclusion of participants with values outside the 3 SD range, participants with complete genetic and phenotypic information were 5,475 for Hb, 5,466 for Hct, 5,405 for MCV, 5,378 for MCHC, 5,413 for MCH, and 5,523 for RBC. Genotyping was conducted using the Illumina 550K array among self‐reported Caucasian individuals, and succeeded in 6,240 individuals (sample call rate ≥ 97.5%). We excluded subjects for excess autosomal heterozygosity, mismatch between called and phenotypic gender, or being outliers identified by the IBS clustering analysis. The final population for genetic analysis comprised 5,974 subjects. SNPs were excluded for minor allele frequency ≤1%, Hardy‐Weinberg equilibrium *P* value<10‐6, or SNP call rate ≤90% resulting in data on 530683 SNPs.

Imputation. In all studies, genotypes were imputed to approximately 2.5 million autosomal SNPs in HapMap, using the Phase II CEU individuals as a reference panel. For imputation software,ARIC, and RS used MACH (http://www.sph.umich.edu/ csg/abecasis/MACH), SNP imputation inferred genotypes probabilistically according to shared haplotype stretches between each study’s samples and HapMap release 22 build 36.

Association analysis. Genome‐wide analyses were conducted within each cohort. Using an additive genetic model, we used linear regression to evaluate the association between the allele dosage and the trait of interest and to quantify the regression slope (β) and standard error (SE(β)). We adjusted all analyses for age and sex (http://www.sph.umich.edu/csg/abecasis/ Metal/index.html).

**References**

Traglia M et al. (2009) Heritability and Demographic Analyses in the large isolated population of Val Borbera suggest advantages in mapping complex traits genes. PLoS One 10: e7554

Richards JB et al. (2008) Bone mineral density, osteoporosis, and osteoporotic fractures: a genome-wide association study. Lancet 371: 1505–1512.

Soranzo N et al. (2009) Meta-analysis of genome-wide scans for human adult stature identifies novel Loci and associations with measures of skeletal frame size. PLoS Genet. 2009 Apr;5(4):e1000445.

Teo YY, Inouye M, Small KS, Gwilliam R, Deloukas P, et al. (2007) A genotype calling algorithm for the Illumina BeadArray platform. 23: 2741–2746.

Howie B., Donnelly P., Marchini J. (2009) A Flexible and Accurate Genotype Imputation Method for the Next Generation of Genome-Wide Association Studies. PLoS Genetics 5(6): e1000529.
