# Supplementary material for: Genome Wide Association Analysis of a Founder Population Identified TAF3 as a Gene for MCHC in Humans
Source: PLoS One. 2013 Jul 31;8(7):e69206. doi: 10.1371/journal.pone.0069206 (PMC3729833; doi:10.1371/journal.pone.0069206)
Supplement: Table S1 — Primers for gene expression analysis in MEL cells. (DOC) [file pone.0069206.s005.doc]

**Table S1**

Primers for gene expression analysis in MEL cells:

specA F 5’-CTGAGCTGAGCAGAGATGGA-3’;

specA R 5’-TTTAACACCTCCGCTCTTCG-3’;

Primers for gene expression analysis in K562 cells:

SPTA1 F 5’-CATGAGCCCAAGTTTGAGAAG-3’;

SPTA1 R 5’-GCAGTATAGTCCTCCAGTGAGACA-3’;

TAF3 F 5’-GTGTGACGACTGCGATGACT-3’

TAF3 R 5’-AGAACCACTGCATCTCTTCTGG-3’

GAPDH F 5’-agggctgcttttaactctggt-3’

GAPDH R 5’-CCCCACTTGATTTTGGAGGGA-3’
