# Supplementary material for: Genome Wide Association Analysis of a Founder Population Identified TAF3 as a Gene for MCHC in Humans
Source: PLoS One. 2013 Jul 31;8(7):e69206. doi: 10.1371/journal.pone.0069206 (PMC3729833; doi:10.1371/journal.pone.0069206)
Supplement: Table S2 — Primers for ChIP analysis in K562 cells. (DOC) [file pone.0069206.s006.doc]

**Table S2**

Primers for ChIP analysis in K562 cells

SPTA1 F 5’-TGAGTGGCTGGCTTATACCC-3’ ;

SPTA1 R 5’-CAGCTCTCTTCACCCCAAGA –3’;
