# Supplementary material for: Genome Wide Association Analysis of a Founder Population Identified TAF3 as a Gene for MCHC in Humans
Source: PLoS One. 2013 Jul 31;8(7):e69206. doi: 10.1371/journal.pone.0069206 (PMC3729833; doi:10.1371/journal.pone.0069206)
Supplement: Supplementary file 5 [file pone.0069206.s007.doc]

Table S3. Comparison of the GWAS results of the present study with previous studies

Val Borbera

Trait	Gene	Variant	Chr	Position	status	n	min_all	MAF	beta	sebeta	P
Hb	PRKCE	rs10495928	2	46206670	observed	1649	G	0.30	-0.1490	0.0424	**4.45E-04**
Hct	PRKCE	rs10168349	2	46214411	imputed	1649	C	0.30	-0.3914	0.1216	**1.29E-03**
Hct	HK1	rs16926246	10	70763398	imputed	1649	T	0.08	0.5437	0.2402	**2.36E-02**
MCH	SLC17A3	rs1408272	6	25950930	imputed	1632	G	0.07	0.0161	0.0043	**1.70E-04**
MCH	HBS1L/MYB	rs7776054	6	135460609	imputed	1632	G	0.24	0.0103	0.0022	**3.99E-06**
MCH	TMPRSS6	rs2413450	22	35800170	imputed	1632	T	0.46	-0.0099	0.0020	**4.28E-07**
MCHC	SPTA1	rs857721	1	156879172	imputed	1655	A	0.26	-0.0029	0.0013	**2.42E-02**
MCHC	CDT1	rs837763	16	87381230	observed	1655	C	0.46	0.0041	0.0011	**4.08E-04**
MCHC	TMPRSS6	rs855791	22	35792882	observed	1655	A	0.45	-0.0028	0.0012	**1.83E-02**
MCV	BCL11A	rs2540917	2	60462263	imputed	1639	C	0.35	0.0057	0.0018	**1.32E-03**
MCV	THRB	rs9310736	3	24325815	imputed	1639	A	0.36	-0.0042	0.0017	**1.58E-02**
MCV	HFE	rs1800562	6	26201120	observed	1639	A	0.06	0.0098	0.0035	**5.29E-03**
MCV	BYSL-CCND3	rs11970772	6	42033268	imputed	1639	A	0.21	0.0058	0.0021	**5.27E-03**
MCV	CD164	rs9374080	6	109723113	imputed	1639	C	0.44	0.0043	0.0018	**1.63E-02**
MCV	HBS1L-MYB	rs4895441	6	135468266	imputed	1639	G	0.26	0.0088	0.0019	**3.40E-06**
MCV	CITED2	rs643381	6	139881116	imputed	1639	A	0.49	-0.0045	0.0017	**8.80E-03**
MCV	IKZF1	rs12718597	7	50395922	imputed	1639	A	0.41	0.0058	0.0017	**5.31E-04**
MCV	MARCH8	rs11239550	10	45344735	imputed	1639	G	0.29	0.0044	0.0019	**2.20E-02**
MCV	TMPRSS6	rs2413450	22	35800170	imputed	1639	T	0.46	-0.0077	0.0017	**8.77E-06**
RBC	PRKCE	rs10495928	2	46206670	observed	1652	G	0.30	-0.0127	0.0035	**2.76E-04**
RBC	HBS1L-MYB	rs9483788	6	135477194	imputed	1652	C	0.25	-0.0125	0.0038	**9.38E-04**

Other studies

Trait	n	min_all	MAF	beta	sebeta	P	Ref
Hb	24167	G	0.34	-0.0744	0.0121	7.58E-10	Ganesh et al.
Hct	24167	C	0.34	-0.2006	0.0286	2.17E-12	Ganesh et al.
Hct	24167	T	0.11	0.3370	0.0513	4.99E-11	Ganesh et al.
MCH	24167	G	0.03	0.0134	0.0015	1.37E-18	Ganesh et al.
MCH	24167	G	0.22	0.0092	0.0008	1.98E-33	Ganesh et al.
MCH	24167	T	0.38	-0.0060	0.0007	8.82E-17	Ganesh et al.
MCHC	24167	A	0.32	-0.0022	0.0004	3.414E-09	Ganesh et al.
MCHC	14377	C	0.38	0.0880	0.0120	3.55E-13	Kamatani et al.
MCHC	11908	A	0.42	-0.0900	0.0100	5.50E-10	Chambers et al.
MCV	24167	C	0.43	0.0031	0.0005	2.13E-11	Ganesh et al.
MCV	14364	A	0.26	-0.0740	0.0130	3.33E-08	Kamatani et al.
MCV	24167	A	0.04	0.0115	0.0011	1.43E-27	Ganesh et al.
MCV	13943	A	0.15	0.5750	0.0650	7.00E-19	Soranzo et al.
MCV	24167	C	0.38	0.0026	0.0005	3.70E-08	Ganesh et al.
MCV	24167	G	0.23	0.0080	0.0005	1.00E-57	Ganesh et al.
MCV	24167	A	0.49	-0.0039	0.0005	2.66E-18	Ganesh et al.
MCV	24167	A	0.28	0.0032	0.0005	8.14E-12	Ganesh et al.
MCV	24167	G	0.23	0.0028	0.0005	1.87E-08	Ganesh et al.
MCV	24167	T	0.38	-0.0054	0.0005	1.08E-30	Ganesh et al.
RBC	14392	G	0.17	-0.0870	0.0160	3.81E-08	Kamatani et al.
RBC	24167	C	0.23	-0.0149	0.0019	1.26E-14	Ganesh et al.

[illegible]
